# P01-049 – Assessment of vascular function in systemic JIA

**DOI:** 10.1186/1546-0096-11-S1-A52

**Published:** 2013-11-08

**Authors:** B Sozeri, K Ozdemir, S Mir

**Affiliations:** 1 Pediatric Nephrology, Ege University, Izmir, Turkey; 2 Pediatric Rheumatology, Ege University, Izmir, Turkey

## Introduction

An increased incidence of cardiovascular disease has been found in rheumatic disorders. Juvenile idiopathic arthritis (JIA) is the most common chronic rheumatic disease in children. Prolonged immunological inflammatory process leads in these patients to an early onset of atherosclerosis.

## Objectives

We aimed to assess the presence of early vascular dysfunction in patients with systemic onset juvenile idiopathic arthritis (SoJIA) and investigate the role of therapy SoJIA in vascular health.

## Methods

Eighteen patients ( 12 males, 6 females) with diagnosis of SoJIA according to the International League of Associations for Rheumatology criteria were compared to 75 age- and sex-matched controls. No participant was overweight, obese, or had a history of hypertension, dyslipidemia, diabetes mellitus, or cardiovascular disease. Arterial stiffness (As) was evaluated by measurement of carotid-femoral pulse wave velocity (PWV) and augmentation index (AIx) with Vicorder.

## Results

The mean age onset of disease was 80,4±28,7 months (range 36-122months). The mean duration of disease and active disease was 79 ± 45 months (range 6–162 months) and 58 ± 49 months (range 1–161 months), respectively.

Patients with SoJIA presented a higher mean PWV and AIx than in controls [(6.16±1.45 m/s vs 5.19±0.63 m/s,P =0.01)and (14.7 ± 8.1%vs 10.4 ± 7.35%,P =0.02)]. Eight (44%) patients with JIA had active disease at study entry. The highest levels of PWV and Aix were found in active patients. Six patients had been macrophage activation syndrome at presentation. In these patients, vascular changes higher than other patients (6.30±0.42 m/s vs 5.17±0.55 m/s,P =0.01, respectively). The corticosteroid therapy was found associated with higher PWV, (P< 0.05), while there was not different between vascular parameters and used non steroid therapy (NSAIDs, MTX, or anti-TNF agents). We also find statistically significant correlation between PWV and disease duration (p = 0.003, r = 0.45).

## Conclusion

Vascular function is impaired in patients with SoJIA at a very young age. Vascular dysfunction may be partly attributed to the effects of disease-related characteristics (inflammation, disease activity, and medications).

## Disclosure of interest

None declared

